# Real-World Experience with Brolucizumab Compared to Aflibercept in Treatment-Naïve and Therapy-Refractory Patients with Diabetic Macular Edema

**DOI:** 10.3390/jcm13061819

**Published:** 2024-03-21

**Authors:** Anne Rübsam, Leopold Hössl, Saskia Rau, Alexander Böker, Oliver Zeitz, Antonia M. Joussen

**Affiliations:** 1 Department of Ophthalmology, Charité—Universitätsmedizin Berlin, 13353 Berlin, Germany; 2 Berlin Institute of Health at Charité—Universitätsmedizin Berlin, Charitéplatz 1, 10117 Berlin, Germany; 3 Smile Eyes Group, 06217 Merseburg, Germany

**Keywords:** brolucizumab, aflibercept, diabetic macular edema, central retinal thickness, best corrected visual acuity

## Abstract

**Background**: To report on the outcome of intravitreal brolucizumab compared to aflibercept in patients with diabetic macular edema (DME). **Methods**: Prospective, observational, study in 35 eyes of 24 patients with a loading dose of five injections of 6 mg brolucizumab every 6 weeks (q6w, treatment-naïve eyes) or a minimum of two injections of brolucizumab q6w after the switch (recalcitrant DME eyes), followed by a treat and extend (T&E) regimen. The results were compared with 40 eyes of 31 DME patients who were treated with aflibercept. The data were obtained from the Berlin Macula Registry. The primary outcome measure was the change in best-corrected visual acuity (BCVA) at week 36. Secondary outcome measures were the change in central retinal thickness (CRT) and the treatment intervals until week 36. **Results**: BCVA increased significantly in treatment-naïve DME eyes treated with either brolucizumab (+0.12 logMAR, +6.4 letters, *p* = 0.03) or aflibercept (+0.19 logMAR, +9.5 letters, *p* = 0.001). In recalcitrant DME eyes, BCVA also increased significantly after switching to brolucizumab (+0.1 logMAR, +5 letters, *p* = 0.006) or aflibercept (+0.11 logMAR, +5.5 letters, *p* = 0.02). All treatment-naïve and recalcitrant DME eyes had a significant decrease in CRT after treatment with brolucizumab (*p* = 0.001 and *p* < 0.001) or aflibercept (*p* = 0.0002 and *p* = 0.03). At week 36, the mean treatment interval for brolucizumab was 11.3 weeks, while for aflibercept, it was 6.5 weeks for treatment-naïve eyes and 9.3 weeks vs. 5.3 weeks for pretreated eyes. **Conclusions**: In routine clinical practice, patients with treatment-naïve and recalcitrant DME showed a favorable response to brolucizumab and aflibercept therapy, with a reduced injection frequency after brolucizumab treatment.

## 1. Introduction

Diabetic macular edema (DME) is a common microvascular complication in patients with diabetes and has become the leading cause of vision loss in the working age population [1]. With the currently available anti-VEGF agents, intensive treatment is often required to dry the macula as much as possible to achieve optimal treatment outcomes. However, patients with DME often have a high medical burden due to multiple comorbidities, and real-world evidence shows that this can lead to high rates of non-adherence, under-treatment of DME, and in turn lower visual acuity gains [2,3]. Therefore, additional treatment options are needed to improve response rates and reduce treatment burden by reducing the frequency of injections and monitoring visits, while preserving visual function in patients with DME. Brolucizumab (Beovu), a single-chain antibody fragment (scFv) with high affinity for VEGF, has a low molecular weight (26 kDa) that allows for more drug per injection compared to other available anti-VEGFs and offers the potential for more effective tissue penetration and longer duration of action [4,5].

One year ago, the KESTREL and KITE pivotal trials of brolucizumab in patients with diabetic macular edema were published [6,7]. After a matching phase of five loading doses every 6 weeks, patients in the brolucizumab group were treated on a q12-week interval, unless they showed disease activity, in which case, treatment was adjusted to a permanent q8-week interval. The comparator drug aflibercept was injected in five doses every 4 weeks followed by fixed q8w dosing. At the end of both studies, the primary endpoint of non-inferiority in the best-corrected visual acuity (BCVA) change from baseline at week 52 of brolucizumab 6 mg vs. aflibercept was met, with >50% of brolucizumab 6 mg subjects being maintained on a q12w interval through week 52 [6].

However, it is well known that results from clinical trials in well-defined patient populations do not necessarily guarantee efficacy in diverse patient populations in real-world clinical settings [8,9,10]. For example, the Protocol T extension study showed that visual gains achieved in RCTs were not maintained with the subsequent standard of care [11].

Furthermore, despite the overall anatomical and functional improvements achieved with anti-VEGF treatment, some patients continue to have persistent DME, despite continuous therapy. A post hoc analysis of the DRCR.net study, Protocol I, estimated that of eyes treated with ranibizumab injections every 4 weeks and then pro re nata (PRN) who have persistent DME at 24 weeks, approximately 40% will have chronic persistent DME at 3 years [12].

Currently the literature on the real-world experience with brolucizumab in treatment-naïve patients with DME is sparse. Additionally, there is a lack of real-world data comparing the efficacy of brolucizumab to aflibercept in DME patients who have previously received anti-VEGF therapy, with a follow-up time of more than one month after the switch. This study aims to evaluate the short-term outcomes of brolucizumab and aflibercept therapy in treatment-naïve DME patients. We also evaluated the effectiveness and safety of intravitreal brolucizumab and intravitreal aflibercept in patients with chronic, therapy-refractory DME, who were already treated for diabetic macular edema and were switched to brolucizumab or aflibercept.

## 2. Materials and Methods

In this institutional, observational, prospective study, 12 eyes of 8 patients with new-onset DME and 23 eyes of 16 patients with recalcitrant DME were treated with brolucizumab between July 2022 and December 2023 at the Department of Ophthalmology, Charité, Universitätsmedizin Germany.

All investigations and measurements adhered to the tenets of the Declaration of Helsinki; the local ethics committee approved the study (EA2/135/22). Informed consent was obtained from patients prior to study enrollment.

We compared the results of aforementioned brolucizumab-treated eyes with 17 eyes of 13 treatment-naïve DME patients and 23 eyes of 18 therapy-refractory DME patients who were switched to aflibercept. For the retrospective cohort analysis, treatment-naïve DME eyes with a full upload of 6 q4w 2 mg aflibercept injections and who were followed-up for at least 36 weeks were included. Furthermore, the outcomes of recalcitrant DME patients, who were switched to aflibercept therapy and had received at least 2 consecutive aflibercept injections after the switch, with a follow-up of at least 36 weeks are reported. The medical record data were extracted and stored into a separate clinical trial database, known as the Berlin Macula Registry, as previously published [13].

The inclusion criteria were patients aged 18 years or older with type 1 or type 2 diabetes mellitus and new-onset center-involving diabetic macular edema requiring treatment with anti-VEGF agents (treatment-naïve group). A center-involving DME was defined as any fluid (intra- and/or subretinal fluid) within a circle with a radius of 1 mm, centered on the fovea on the EDTRS map in the Heidelberg software of the OCT device (software version 6.9.5.0, Heidelberg Spectralis, Heidelberg Engineering, Heidelberg, Germany). In addition, DME patients with persistent center-involving DME despite at least 6 months of anti-VEGF treatment with bevacizumab, ranibizumab, and/or aflibercept were included (switch group).

Exclusion criteria were macular edema of non-DME origin, proliferative membranes that could cause tractional retinal detachment or any ocular condition that could interfere with potential visual improvement (i.e., dense cataract, amblyopia), signs of any other active retinal disease in the study eye, especially signs of an active uveitis, patients with status post-uveitis, and poor image quality.

### 2.1. Baseline

At baseline, 12 eyes of 8 patients were treatment-naïve and 23 eyes of 16 patients were switched to brolucizumab. Furthermore, medical data of 17 eyes of 13 treatment-naïve DME patients treated with aflibercept and 23 eyes of 18 patients with a therapy switch to aflibercept were recorded. Baseline diagnostic procedures included a BCVA assessment using logarithm of the minimum angle of resolution (logMAR, brolucizumab group) converted to logMAR from decimal (aflibercept group), anterior and posterior segment examination, and SD-OCT (Heidelberg Spectralis, Heidelberg Engineering, Heidelberg, Germany). In the treatment-naïve group, fluorescein angiography (FA, Heidelberg Spectralis, Heidelberg Engineering, Heidelberg, Germany) was performed at baseline to identify peripheral and severe macular ischemia and active neovascularization.

### 2.2. Treatment and Follow-Up

In the brolucizumab arm, treatment-naïve patients were treated with a modified treat and extend (T&E) protocol, similar to the KITE and KESTREL protocols, with 5 injections of 6 mg brolucizumab at 6-week intervals (upload). For the first injection of the maintenance phase (week 36), brolucizumab was administered at a minimum of an 8-week interval in all patients, as this is the minimum interval recommended for brolucizumab after the upload according to professional medical information. Patients were treated with q8w dosing in case of (a) improved, but still significant center-involving DME, (b) in case of worsening, or (c) persistent center-involving DME at week 24. Patients were treated with q10w dosing in case of resolved center-involving DME at week 24 (time point of the 5th injection of the upload). Patients were treated with q12w dosing, if the center-involving DME resolved already earlier (already at the prior visits at week 6, 12, or 18). At week 36, patients were extended in 2-weekly intervals if there was further no disease activity (DA). Patients continued q8w dosing or treatment intervals were shortened in 2-week intervals in case of DA (persistent or worsened center-involving DME). In the aflibercept arm, patients have been treated with 6 injections of 2 mg aflibercept at 4-week intervals (upload) and with a T&E protocol afterwards with a minimum treatment interval of 4 weeks.

In the switch group, pretreated patients were switched to 6 mg brolucizumab if intra- and/or subretinal fluid persisted despite at least 6 months of treatment including at least 6 consecutive monthly anti-VEGF injections with aflibercept, ranibizumab, bevacizumab, and/or at least one injection of a dexamethasone implant. A minimum of 2 injections q6w were performed after the switch to brolucizumab, after which patients were kept at q6w (for a maximum of 5 injections = full upload) as needed until the center-involving edema resolved. Thereafter, patients were treated according to the T&E protocol as aforementioned. In the aflibercept arm, pretreated eyes with a minimum of 2 q4w aflibercept injections after the switch and a follow-up of at least 36 weeks after the switch were included.

We evaluated clinical parameters including SD-OCT during the upload at each brolucizumab injection (=baseline, week 6, 12, 18, 24) and 8–12 weeks later (week 36) and at each aflibercept injection (=baseline, week 4, 8, 12, 16, 20) and thereafter every 4 weeks until week 36.

### 2.3. OCT Data

Standard OCT settings were 20° × 20° volume scan, 49 sections at a distance of 122 μm. Central retinal thickness (CRT) in µm was automatically calculated by the device software (software version 5.1.2.0) as the distance from the retinal pigment epithelium (RPE) to the inner limiting membrane (ILM) at the highest point within a circle of 1 mm radius circle centered on the fovea. We also assessed whether the DME was center-involving, defined as the presence of SRF or IRF within a circle of 1 mm circle centered on the fovea on the EDTRS grid and the presence of intra- or subretinal fluid.

### 2.4. Main Outcome Measures

The primary outcome measure was defined as the change in BCVA at week 36. Secondary outcome measures were the change in CRT, assessed by SD-OCT, safety, percentage of eyes achieving a resolution of the center-involving DME. Furthermore, the status of fluid (subretinal and/or intraretinal), the number of injections until week 36, and the treatment intervals after the upload and the need for a full upload in switch patients were evaluated.

#### Statistical Analysis

Data exploration for normal distribution was performed using a Shapiro-Wilk test. Means ± SEM and statistically significant differences are reported. Means of CRT and BCVA at baseline were compared to follow-up visits (week 6–week 36) using a Wilcoxon signed rank test to account for a non-Gaussian distribution. A *p*-value less than 0.05 was considered significant. Statistical analysis was performed using GraphPad Prism (GraphPad Software, version 9.5.1, San Diego, CA, USA).

## 3. Results

### 3.1. Patients’ Characteristics

In the brolucizumab group, 12 eyes of 8 treatment-naïve patients (6 male and 2 female) with center-involving DME and a mean age of 55 ± 19 years were included in this study. According to FA, four patients were treated with additional panretinal laser treatment (PRP), if retinal neovascularization or severe ischemia were present, while laser treatment was deferred until after the first injection. The distribution of diabetic retinopathy severity was 16.5%, 50%, 16.5%, and 16.5% for nonproliferative diabetic retinopathy (NPDR) with PRP, NPDR without PRP, proliferative diabetic retinopathy (PDR) with PRP, and PDR without PRP, respectively. In the aflibercept group, 17 eyes of 13 treatment-naïve patients (9 male and 4 female) with center-involving DME and a mean age of 64 ± 19 years were included in this study. Eight patients were treated with deferred additional panretinal laser treatment. The distribution of diabetic retinopathy severity was 12%, 47%, 35%, and 6% for NPDR with PRP, NPDR without PRP, PDR with PRP, and PDR without PRP.

In addition, a total of 23 eyes of 16 pretreated DME patients (11 male and 5 female) with center-involving DME and a mean age of 60 ± 8 years (switch group) were included in the brolucizumab group. Two patients were treated with panretinal laser treatment during the study period. The distribution of diabetic retinopathy severity was 17%, 56%, 22%, and 5% for NPDR with PRP, NPDR without PRP, PDR with PRP, and PDR without PRP, respectively. None of the patients received focal macular laser treatment. Patients were treated with multiple IVI with different intravitreal agents before the switch. Previous injections were bevacizumab in 21 patients, ranibizumab in 9 patients, aflibercept in 6 patients, and a dexamethasone implant in 9 patients. Patients had received a mean of 18 (range 7–32) injections prior to the switch. The reason for the switch was a persistent center-involving DME in all 23 patients. For the aflibercept group, the medical data of 23 eyes of 18 pretreated DME patients (13 male and 5 female) with center-involving DME and a mean age of 67 ± 9 years (switch group) were extracted. Six patients were treated with panretinal laser treatment during the study period. The distribution of diabetic retinopathy severity was 17%, 22%, 48%, and 13% for NPDR with PRP, NPDR without PRP, PDR with PRP, and PDR without PRP, respectively. None of the patients received focal macular laser treatment. Previous injections were bevacizumab in 15 patients, ranibizumab in 16 patients, a dexamethasone implant in 5 patients, and a fluocinolone implant in one patient. Patients had received a mean of 12 (range 6–48) injections prior to the switch. The reason for the switch was a persistent center-involving DME in all 23 patients. A summary of all patient characteristics is shown in Table 1.

### 3.2. Change in Best-Corrected Visual Acuity

#### 3.2.1. Treatment-Naïve Eyes

**Figure 1:**
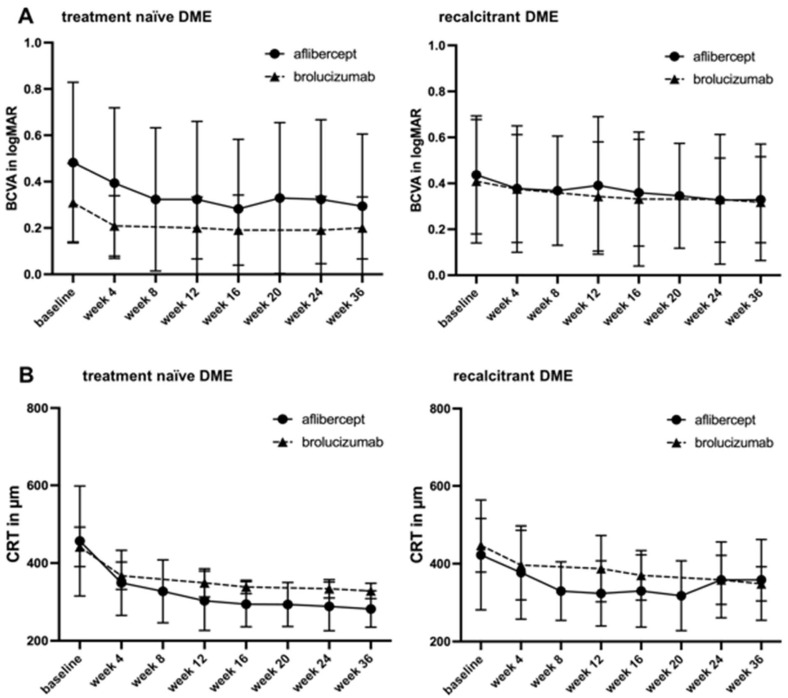
Changes in best corrected visual acuity (BCVA) and central retinal thickness (CRT) after therapy with brolucizumab or aflibercept in treatment-naïve and recalcitrant patients with diabetic macular edema (DME). Figure shows the difference in change in (**A**) BCVA in logMAR and (**B**) CRT in µm after 5 injections of brolucizumab at 6-week intervals and during the maintenance phase 12 weeks later (week 36) or after 6 injections of aflibercept at 4-week intervals and during the maintenance phase until week 36.

Mean BCVA increased significantly in brolucizumab-treated eyes from baseline to the last visit from 0.31 ± 0.1 logMAR to 0.18 ± 0.1 logMAR (*p* = 0.03; for details, see Table 2, Figure 1A). In detail, the mean BCVA increased significantly after each injection (*p* = 0.01 after the first injection, *p* = 0.03 after the second injection, *p* = 0.007 after the third injection, *p* = 0.03 after the fourth injection, and *p* = 0.03 after the fifth injection at week 36).

Mean BCVA increased significantly in aflibercept-treated eyes from baseline to the last visit from 0.48 ± 0.3 logMAR to 0.29 ± 0.3 logMAR (*p* = 0.001; for details, see Table 2, Figure 1A). In detail, the mean BCVA increased after each injection but without statistical significance after the first injection and statistically significant thereafter (*p* = 0.06 after the first injection, *p* = 0.01 after the second injection, *p* = 0.04 after the third injection, *p* = 0.005 after the fourth injection, *p* = 0.001 after the fifth injection, *p* = 0.001 after the sixth injection, and *p* = 0.001 at week 36). 

#### 3.2.2. Therapy-Switch Eyes

In the brolucizumab group, the mean BCVA improved significantly from baseline to the last visit from 0.43 ± 0.3 logMAR to 0.33 ± 0.2 logMAR (*p* = 0.006; for details, see Table 2, Figure 1A). In detail, the mean BCVA increased without statistical significance after the first injection and with statistical significance thereafter (*p* = 0.75 after the first injection, *p* = 0.02 after the second injection, *p* = 0.01 after the third injection, *p* = 0.01 after the fourth injection, and *p* = 0.006 after the fifth injection).

For aflibercept-treated eyes, the mean BCVA improved significantly from baseline to the last visit from 0.44 ± 0.2 logMAR to 0.33 ± 0.1 logMAR (*p* = 0.02; for details, see Table 2, Figure 1A). In detail, the mean BCVA increased without statistical significance until after the fourth injection and with statistical significance thereafter (*p* = 0.14 after the first injection, *p* = 0.16 after the second injection, *p* = 0.20 after the third injection, *p* = 0.07 after the fourth injection, *p* = 0.03 after the fifth injection, *p* = 0.01 after the sixth injection, and *p* = 0.02 at week 36).

### 3.3. Change in Central Retinal Thickness

#### 3.3.1. Treatment-Naïve Eyes

Mean CRT decreased significantly in brolucizumab-treated eyes from baseline to the last visit from 441.8 ± 50.9 µm to 328.4 ± 19.8 µm (*p* = 0.001; for details, see Table 2, Figure 1B). In detail, the mean CRT decreased significantly after each injection (*p* = 0.002 after the first injection, *p* = 0.0005 after the second injection, *p* = 0.002 after the third injection, *p* = 0.001 after the fourth injection, and *p* = 0.001 after the fifth injection at week 36).

For aflibercept-treated eyes, the mean CRT decreased significantly from baseline to the last visit from 456.7 ± 50.9 µm to 281.7 ± 46.8 µm (*p* = 0.0002; for details, see Table 2, Figure 1B). In detail, the mean CRT decreased significantly after each injection (*p* = 0.001 after the first injection, *p* = 0.002 after the second injection, *p* = 0.0009 after the third injection, *p* = 0.001 after the fourth injection, *p* = 0.003 after the fifth injection, *p* = 0.003 after the sixth injection, and *p* = 0.0002 at week 36).

#### 3.3.2. Therapy-Switch Eyes

In the brolucizumab group, the mean CRT decreased significantly from baseline to the last visit from 447.1 ± 68.8 µm to 355.8 ± 42.3 µm at week 36 (*p* < 0.0001; for details, see Table 2, Figure 1B). In detail, the mean CRT decreased significantly after each injection (*p* = 0.001 after the first injection, *p* = 0.0002 after the second injection, *p* < 0.0001 after the third injection, *p* < 0.0001 after the fourth injection, and *p* < 0.0001 after the fifth injection).

The mean CRT decreased significantly after therapy switch to aflibercept from baseline to the last visit from 422.4 ± 141.1 µm to 358.6 ± 103.8 µm at week 36 (*p* = 0.03; for details, see Table 2, Figure 1B). In detail, the mean CRT decreased significantly after each injection of the upload and increased slightly thereafter (*p* = 0.01 after the first injection, *p* = 0.009 after the second injection, *p* = 0.0003 after the third injection, *p* = 0.001 after the fourth injection, *p* = 0.001 after the fifth injection, *p* = 0.01 after the sixth injection, and *p* = 0.03 at week 36).

### 3.4. Anatomic Outcome and Treatment Interval

The treatment-naïve patients were either treated with a total of five brolucizumab injections at 6-week intervals or six injections with aflibercept at 4-week intervals. At week 24 (time point of fifth brolucizumab injection), all eyes treated with brolucizumab had reduced fluid compared to the baseline. The proportion of eyes with intraretinal fluid (IRF) was reduced to 50% compared to the baseline. The criterion for extending the treatment interval to q10w or q12w was a resolution of center-involving DME at this time point, which was fulfilled in 83% (10/12 eyes). Thus, the mean treatment interval was extended to 10.8 weeks. At week 36, the proportion of eyes with any IRF decreased further to 43%. The number of eyes with resolved center-involving DME increased to 92% (11/12 eyes) and the treatment intervals were extended to a mean of 11.3 weeks. Until week 36, every patient had received six injections.

In the aflibercept group, all eyes showed a reduction in fluid at week 24, which was 4 weeks after the sixth aflibercept injection. The proportion of eyes with any SRF or IRF decreased to 64.7% (11/17 eyes) compared to the baseline, and the number of eyes with resolution of center-involving DME was 75%. At week 36, there was a further reduction of fluid in seven patients and the proportion of eyes with any IRF or SRF decreased to 58.8% (10/17 eyes). The number of eyes without center-involving DME remained stable at 75%, and the mean treatment intervals were extended to 6.5 weeks. Up to week 36, the mean number of aflibercept injections was 7.5 (range 6–9), with a maximum of 9 injections (q4w) in 35% of eyes.

The recalcitrant DME eyes were treated with a minimum of two brolucizumab injections at 6-week intervals or two aflibercept injections every 4 weeks. After a total of two brolucizumab injections (time point of third injection, week 18), all eyes had reduced fluid. The proportion of eyes with remaining IRF was 96% and the number of eyes with a dry fovea was 22% (5/23 eyes). Therefore, these 22% of eyes were already extended to an q8w dosing. At week 36, after a total of five brolucizumab injections, the proportion of eyes with any IRF decreased to 87% and the number of eyes with a dry fovea increased to 44% (10/23 eyes) and the treatment intervals were extended to a mean of 9.3 weeks. Overall, 69.5% (16/23) of recalcitrant DME eyes required a full upload after the therapy switch, and in 22% of the eyes, the treatment intervals could already be extended after only two brolucizumab injections.

After the therapy switch to aflibercept, after a total of two injections (week 12), 96% of eyes had reduced fluid. The proportion of eyes with remaining IRF or SRF was 96%, and the number of eyes with a resolved center-involving DME was 26% (6/23 eyes). By week 36, the proportion of eyes with any IRF or SRF decreased to 91%, the number of eyes with a dry fovea increased to 34% (8/23 eyes), and the mean treatment interval was extended to 5.3 weeks. After the therapy switch, 52% of eyes required a full upload, and in 26% of eyes, treatment intervals could already be extended after only two aflibercept injections. Until week 36, the mean number of aflibercept injections was 7.2 (range 5–9), with a maximum of 9 injections (q4w) in 13% of eyes. Overall, 56.5% (13/23) of recalcitrant DME eyes required a full upload with aflibercept after the therapy switch.

### 3.5. Safety

In our study, a total of 502 injections were performed. No patient experienced any endophthalmitis, intraocular inflammation (IOI), or vascular occlusive event.

## 4. Discussion

**Figure 2:**
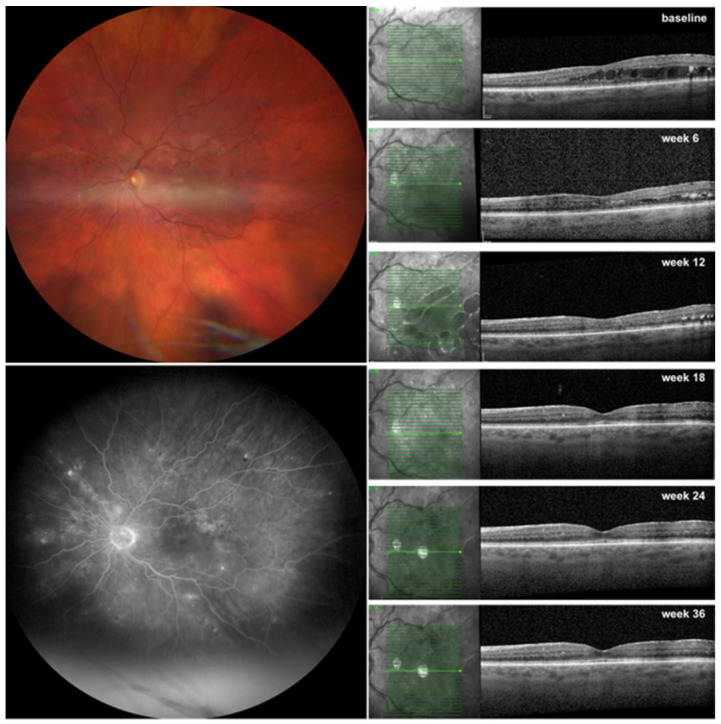
Response to brolucizumab therapy in a treatment-naïve patient with diabetic macular edema (DME) assessed by optical coherence tomography (OCT). Left eye of a 73-year-old woman with severe nonproliferative diabetic retinopathy (NPDR) on fundus photography (upper left panel) and on fluorescein angiography (lower left panel) with intraretinal fluid (IRF) and hyperreflective foci on OCT (right panel) with a resolved center-involving DME after 2 injections q6w (week 12) and a complete regression of IRF after 3 injections (week 18), which was maintained throughout the upload (week 24) and the patient was extended to q12 w (week 36).

**Figure 3:**
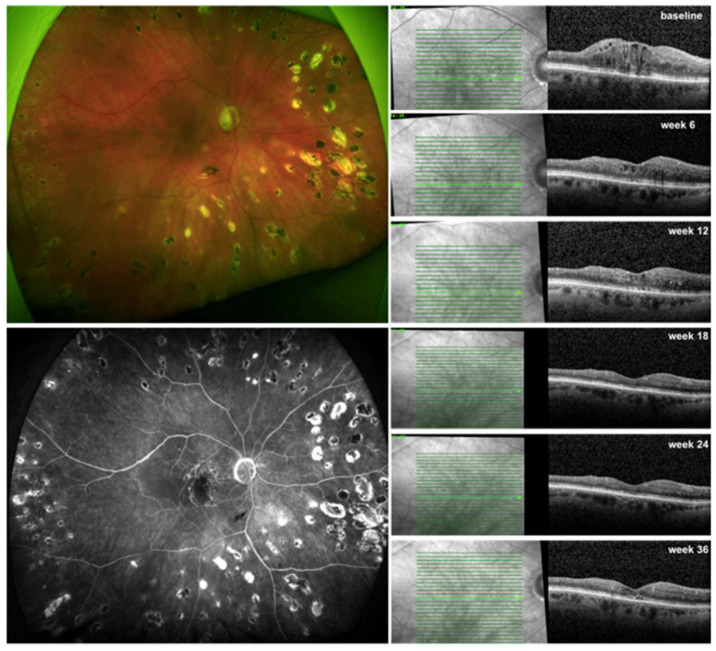
Response to brolucizumab therapy in a therapy-refractory patient with diabetic macular edema (DME) assessed by optical coherence tomography (OCT). Right eye of a 72-year-old woman with proliferative diabetic retinopathy (PDR) on fundus photography (upper left panel) and on fluorescein angiography (lower left panel) with intraretinal fluid (IRF) and disintegration of the external limiting membrane (ELM) on OCT (right panel). Previous intravitreal therapies were 6 injections of bevacizumab, 7 injections of aflibercept, and 4 dexamethasone implants before switching to brolucizumab. A resolved center-involving DME was seen after 3 injections q6w (week 18) and a complete regression of IRF after 4 injections q6w (week 24). The patient was extended to q12w (week 36) with a minimal recurrence of foveal IRF.

In the present study, we prospectively evaluated the efficacy of brolucizumab in treatment-naïve patients with DME. We demonstrated a significant increase in BCVA along with a significant reduction in CRT after 9 months of therapy (Figure 2). Additionally, 92% of eyes experienced the resolution of center-involving edema, and they were maintained on treatment intervals of 10 to 12 weeks. This study also evaluated switching to brolucizumab in recalcitrant DME patients (Figure 3). These patients also showed a significant improvement in BCVA, albeit to a lesser extent, along with a significant decrease in CRT. After switching to brolucizumab, all patients had reduced fluid and 44% of eyes had no residual fluid at the fovea with a mean treatment interval of 9.3 weeks, nine months later. We compared our results with the data extracted from the Berlin Macula Registry on treatment-naïve and recalcitrant DME patients who were treated with aflibercept. These patients experienced a comparable increase in BCVA and a significant reduction in CRT. The main difference was the smaller number of injections required for brolucizumab-treated eyes compared to those treated with aflibercept (6 vs. 7.5 for treatment-naïve patients and 6 vs. 7.2 for pretreated patients).

Our findings are consistent with those in the existing literature. Currently, the only other prospective studies on the comparison of brolucizumab and aflibercept in treatment-naïve DME patients are the two pivotal trials KITE and KESTREL [6,7]. For the primary endpoint of change in VA, we demonstrated a VA gain of +0.12 logMAR, equivalent to +6.4 letters at week 36 for brolucizumab and +0.19 logMAR or +9.5 letters for aflibercept. These results are comparable to the VA gain of +9.2 letters in the brolucizumab 6 mg arm and +10.5 letters in the aflibercept arm (KESTREL), as well as +10.6 letters in the brolucizumab arm compared to +9.4 letters for aflibercept in KITE at week 52 [6]. Furthermore, a retrospective case series of 45 treatment-naïve DME patients treated with either brolucizumab or aflibercept demonstrated a similar increase in BCVA of +12.6 letters for the brolucizumab arm and +12.7 letters for aflibercept arm [14]. Overall, brolucizumab appears to be at least as effective as the comparator drug aflibercept in improving VA in treatment-naïve DME patients, as shown in both randomized clinical trials and routine clinical practice.

Our study also found a smaller but still significant improvement in BCVA for patients who switched to brolucizumab or aflibercept due to recalcitrant DME. The mean change in BCVA was +0.1 logMAR or +5 letters at week 36 for brolucizumab, compared to +0.11 logMAR or +5.5 letters for aflibercept. Currently, there is only one other prospective clinical trial on patients with DME who were switched to brolucizumab compared to aflibercept for chronic DME. The KINGFISHER prospective, randomized, controlled clinical trial reported a VA gain of +12.2 letters for brolucizumab and +11 letters for aflibercept at week 52 [15]. However, the analysis included treatment-naïve DME patients as well, and the dosing regimen was a fixed 4-week dosing for both drugs, which is currently not approved for brolucizumab for the treatment of DME or nAMD [15]. There is only one more retrospective case series on 23 eyes of 20 recalcitrant DME patients treated with brolucizumab, but with a much shorter follow-up of only 1 month after the switch by Hirano et al. [16]. In this study, a BCVA gain of +0.14 logMAR (+7 letters) 4 weeks after a single injection is reported [16]. The overall smaller VA gain compared to treatment-naïve DME eyes, also seen in our study, is consistent with results in the literature on recalcitrant DME patients, where several authors have demonstrated an anatomic benefit with no or only moderate improvement in visual acuity. There appears to be an association between BCVA improvement and changes in the retinal structure by the chronic DME such as the integrity of the external limiting membrane [9], which may be a sign of advanced photoreceptor damage [17,18,19]. To avoid this, an earlier switch may have resulted in better BCVA improvement after the switch.

Our study demonstrated anatomic improvement with a significant reduction in CRT in treatment-naïve DME patients at week 36 (−123 µm for brolucizumab and −175 µm for aflibercept). These results are comparable to the change in CRT observed in KESTREL (−166 μm for brolucizumab 6 mg and −160 μm for aflibercept) and KITE (−197 μm for brolucizumab and −164 μm for aflibercept) at week 52 [6]. The case series by Elhamaky et al. also showed a similar reduction of 160 µm for brolucizumab at week 52 [14]. Along with a significant improvement in CRT, we also demonstrated a notable reduction in fluid, which favors brolucizumab over aflibercept. Throughout the follow-up, all treatment-naïve patients had a lower proportion of fluid at each visit. At week 36, the proportions of patients with IRF were 33% for brolucizumab and 58.8% for aflibercept. In KESTREL, the proportions of subjects with IRF and/or SRF were 60.3% in the brolucizumab 6 mg arm and 73.3% in the aflibercept arm [6]. In KITE, the proportion of subjects with IRF and/or SRF at week 52 was 54.2% in the brolucizumab arm vs. 72.9% in the aflibercept arm [6]. In our study, patients’ treatment intervals were extended after the initial 5 q6w injections, if there was no residual fluid in the fovea. After the upload with brolucizumab, 92% (11/12) of eyes met this disease activity criterion, resulting in a mean treatment interval of 11.3 weeks. At week 36, 75% of eyes treated with aflibercept had no residual fluid at the fovea, with a mean treatment interval of 6.5 weeks. The difference in the number of injections in our study between brolucizumab- and aflibercept-treated eyes is related to the superior anatomic improvement after brolucizumab treatment and to the different dosing schemes of both drugs, with a minimum treatment interval of 8 weeks recommended after the upload for brolucizumab. Thus, the mean number of injections until week 36 was 6 for brolucizumab and 7.5 for aflibercept.

Our study demonstrated significant anatomical improvement even in recalcitrant DME patients who had received numerous injections of different anti-VEGF agents and even steroids before switching to brolucizumab or aflibercept. The mean reduction in CRT was −93 µm for brolucizumab and −63 µm for aflibercept. After only two brolucizumab injections, in 22% (5/23) of eyes, treatment intervals could already be extended. This number increased to 44% (10/23) of eyes at 36 weeks after a total of five injections. In comparison, after only two aflibercept injections, 26% of eyes had a resolution of center-involving DME, and 34% were extended after the complete upload of 6q4w injections. The case series by Hirano et al. showed a similar significant increase in CRT, with a mean reduction of −214.3 µm just 1 month after the switch [16]. Information regarding the exact changes in fluid was not available in this study. However, another case series showed a reduction of CRT and fluid in all three cases, which was consistent over 12 weeks, with a recurrence of fluid in all cases at week 16 [20]. Therefore, similar to our results, an extension of treatment intervals could be achieved in chronic DME patients in particular after switching to brolucizumab compared to aflibercept.

The molecular characteristics of brolucizumab may be one of the reasons for the favorable morphological improvement and prolonged durability observed in this study. Brolucizumab is a novel VEGF inhibitor with a molecular weight of 26 kDa [4]. It is smaller than the commercially available ranibizumab (48 kDa) and aflibercept (97–115 kDa) [4,5]. Brolucizumab can be concentrated to 120 mg/mL due to its high solubility. Therefore, the binding affinity of brolucizumab for VEGF is higher than that of ranibizumab and aflibercept. On the other hand, aflibercept binds related growth factors next to VEGF-A, such as placental growth factors 1 and 2 (PLGF1 and PLGF2) and VEGF-B [21]. We, among others, have shown the beneficial effects of this dual blockade specifically on the inflammatory response in retinal vascular disease, such as in experimental models of nAMD [22]. The significant anatomic response of recalcitrant DME patients to brolucizumab or aflibercept could also be caused by tachyphylaxis to the previous molecule due to neutralizing antibodies, altered surface receptor expression, and/or altered pharmacokinetics.

One particular concern raised with brolucizumab is related to its pro-inflammatory properties, which was first published following the initial approval for the treatment of neovascular age-related macular degeneration (nAMD) [23,24,25]. In our study, we performed 175 brolucizumab intravitreal injections over a period of 9 months without any serious adverse events, in particular, no cases of IOI or vasculitis. Since DME is an inflammatory disease, there has been a concern that IOI may be more common in DME than in controls [26,27]. However, the reported IOI rates were 1.1% and 2.2% for the 6 mg brolucizumab group in KITE and KESTREL [7,28,29]. In addition, the 52-week results from the KINGFISHER clinical trial demonstrated no differences in the safety profile between brolucizumab (IOI rate 4.0%) and aflibercept (IOI rate 2.9%) [15].

Our study has certain limitations such as the, in part, retrospective nature of the study and thus the risk of selection bias. Other limitations are the small number of patients and the relatively short follow-up of only 9 months. First of all, we were interested in the initial treatment response of treatment-naïve and recalcitrant DME patients switching to brolucizumab compared to aflibercept. Furthermore, the loss of follow-up increased dramatically after the 9-month period, so the results would have been less accurate afterwards. Patients had a satisfactory gain in VA and a favorable anatomic outcome, so patients tended to skip later appointments. Moreover, DME patients have a large number of comorbidities and often have many visits to different specialists, with a high rate of interruption of the intravitreal treatment of up to 46% in the first year [2].

## 5. Conclusions

In summary, our real-world experience with brolucizumab and aflibercept in treatment-naïve and therapy-refractory DME patients demonstrates clinically meaningful visual acuity gains and excellent anatomic improvements with a so-far low risk profile at 36 weeks. One main advantage of brolucizumab is the smaller number of injections required to achieve these results. Therefore, brolucizumab may provide an additional therapeutic option in DME, potentially reducing the burden on patients, physicians, and the healthcare system.

## Figures and Tables

**Table 1:** Baseline characteristics of 29 new-onset and 48 eyes with chronic diabetic macular edema treated with brolucizumab or aflibercept.

	Brolucizumab		Aflibercept	
	Treatment-Naïve DME	Recalcitrant DME	Treatment -Naïve DME	Recalcitrant DME
No. of eyes (patients)	12 (8)	23 (16)	17 (13)	23 (18)
Male/female	6/2	11/5	9/4	13/5
Age, years	55.3 (±19)	60.2 (±8)	64.5 (±19)	67.4 (±9)
Diabetic retinopathy, %				
Mild NPDR:	-	9	12	11
Moderate NPDR:	8	9	12	5
Severe NPDR:	33	52	35	17
PDR:	59	30	41	67
No. of eyes with prior vitrectomy	0/12	1/23	0/17	1/23
No. of eyes with prior PRP	0/12	9/23	3/17	16/23
No. of eyes with prior intravitreal	N/A		N/A	
Bevacizumab		21		15
Ranibizumab		9		16
Aflibercept		6		N/A
Dexamethasone		6		5
BCVA (logMAR)	0.31 ± 0.1	0.43 ± 0.3	0.48 ± 0.3	0.44 ± 0.2
CRT, µm	441.8 ± 50.9	447.1 ± 68.8	456.7 ± 141.5	422.4 ± 141.1
Intraretinal fluid (%)	100%	100%	100%	100%
Subretinal fluid (%)	0%	0%	17%	26%

BCVA = best-corrected visual acuity; CRT = central retinal thickness; logMAR = logarithm of minimal angle of resolution; N/A = not applicable; NPDR = non proliferative diabetic retinopathy; PDR = proliferative diabetic retinopathy.

**Table 2:** Mean change in best-corrected visual acuity (BCVA) and central retinal thickness (CRT) in 29 new-onset and 48 eyes with chronic diabetic macular edema treated with brolucizumab or aflibercept at baseline and during follow-up.

	Treatment-Naïve DME				Recalcitrant DME			
Visit	Mean BCVA + SD (logMAR) *p*-Value *		Mean CRT + SD (µm) *p*-Value *		Mean BCVA + SD (logMAR) *p*-Value *		Mean CRT + SD (µm) *p*-Value *	
Anti-VEGF	Brolucizumab	Aflibercept	Brolucizumab	Aflibercept	Brolucizumab	Aflibercept	Brolucizumab	Aflibercept
Baseline	0.31 ± 0.1	0.48 ± 0.3	441.8 ± 50.9	456.7 ± 141.5	0.43 ± 0.3	0.44 ± 0.2	447.1 ± 68.8	422.4 ± 141.1
Week 4–6	0.21 ± 0.1 *p* = 0.01	0.39 ± 0.3 *p* = 0.06	367.2 ± 35.1 *p* = 0.002	349.1 ± 83.7 *p* = 0.001	0.39 ± 0.3 *p* = 0.75	0.38 ± 0.2 *p* = 0.14	406.7 ± 121.0 *p* = 0.001	377.1 ± 119.8 *p* = 0.01
Week 8	N/A	0.32 ± 0.3 *p* = 0.01	N/A	327.1 ± 81.0 *p* = 0.002	N/A	0.37 ± 0.2 *p* = 0.16	N/A	329.7 ± 75.4 *p* = 0.009
Week 12	0.20 ± 0.1 *p* = 0.03	0.32 ± 0.3 *p* = 0.04	349.1 ± 35.1 *p* = 0.0005	303.0 ± 76.3 *p* = 0.0009	0.35 ± 0.2 *p* = 0.02	0.39 ± 0.3 *p* = 0.20	387.3 ± 85.2 *p* = 0.0002	323.7 ± 83.7 *p* = 0.0003
Week 16–18	0.19 ± 0.1 *p* = 0.007	0.28 ± 0.3 *p* = 0.005	338.5 ± 17.1 *p* = 0.002	294.0 ± 58.1 *p* = 0.001	0.35 ± 0.3 *p* = 0.01	0.36 ± 0.2 *p* = 0.07	370.2 ± 63.6 *p* < 0.0001	329.8 ± 93.1 *p* = 0.001
Week 20	N/A	0.32 ± 0.3 *p* = 0.001	N/A	293.4 ± 56.8 *p* = 0.003	N/A	0.34 ± 0.2 *p* = 0.03	N/A	317.7 ± 89.8 *p* = 0.001
Week 24	0.19 ± 0.1 *p* = 0.03	0.31 ± 0.3 *p* = 0.001	333.7 ± 23.7 *p* = 0.001	288.5 ± 62.7 *p* = 0.003	0.34 ± 0.3 *p* = 0.001	0.33 ± 0.1 *p* = 0.01	356.7 ± 66.8 *p* < 0.0001	358.5 ± 97.5 *p* = 0.01
Week 36	0.18 ± 0.1 *p* = 0.03	0.29 ± 0.3 *p* = 0.001	328.4 ± 19.8 *p* = 0.001	281.7 ± 46.8 *p* = 0.0002	0.33 ± 0.2 *p* = 0.006	0.33 ± 0.1 *p* = 0.02	356.8 ± 42.3 *p* < 0.0001	358.6 ± 103.8 *p* = 0.03

BCVA = best corrected visual acuity; CRT = central retinal thickness; logMAR = logarithm of minimal angle of resolution; N/A = not applicable; SD = standard deviation; VEGF = vascular endothelial growth factor; * Wilcoxon signed rank test of visit (week)—baseline.

## Data Availability

Data are available as a Supplemental File.

## Supplementary Materials

The following supporting information can be downloaded at: https://www.mdpi.com/article/10.3390/jcm13061819/s1, Table S1: All patients’ raw data.
